# The COVID-19 pandemic, well-being, and transitions to post-secondary education

**DOI:** 10.1007/s11150-022-09623-9

**Published:** 2022-09-23

**Authors:** Malte Sandner, Alexander Patzina, Silke Anger, Sarah Bernhard, Hans Dietrich

**Affiliations:** 1 Institute for Employment Research (IAB), Nuremberg, Germany; 2 University of Bamberg, Bamberg, Germany; 3 Institute of Labor Economics (IZA), Bonn, Germany

**Keywords:** COVID-19, High school graduates, Mental and physical well-being, Life satisfaction, School-to-work transition, I21, I18, J24

## Abstract

This study examines the immediate and intermediate effects of the COVID-19 pandemic on the well-being of two high school graduation cohorts (2020 and 2021) and how changes in well-being affect students’ educational plans and outcomes. Our unique panel data on 3697 students from 214 schools in 8 German federal states contain prospective survey information on three dimensions of well-being: mental health problems, self-rated health, and life satisfaction. Data is collected several months before (fall 2019), shortly before and soon after (spring 2020) as well as several months after (fall/winter 2020/21) the beginning of the COVID-19 pandemic. Applying difference-in-differences designs, random effect growth curve models, and linear regression models, we find that school closures had a positive immediate effect on students’ well-being. Over the course of the pandemic, however, well-being strongly declined, mainly among the 2021 graduation cohort. We show that a strong decline in mental health is associated with changes in educational and career plans and transition outcomes. As adverse life experiences in adolescence are likely to accumulate over the life course, this study is the first to exhibit potential long-lasting negative effects of the COVID-19 pandemic on education and careers of young individuals.

## Motivation

The COVID-19 pandemic and the related policies to stop the spread of the coronavirus, particularly school closures, present a severe shock to mental and physical well-being for millions of young individuals worldwide. Distancing measures may affect the mental and physical health and life satisfaction of young individuals, as these measures massively change the schooling, learning processes and leisure activities of students, such as physical activity, time spent in front of screens, social contacts, substance use, and sleep time (Belot et al., 2021; Champeaux et al., 2020; Davis, 2021; Hisler & Twenge, 2021; Orgilés et al., 2020; Shanahan et al., 2020). Families with younger kids suffered from decreased well-being (Pisano et al., 2020; Stassart et al., 2021) but pandemic policies may especially impact the well-being of young individuals who are close to graduation because the measures not only affected schooling and leisure but also strongly reduced students’ perceived career security and job and educational opportunities. As students’ well-being presents a crucial resource in the process of educational decision-making and socioeconomic attainment (Haas, 2006), shocks to well-being may disrupt the transition from upper secondary to post-secondary education. Such transition disruptions at this stage may have negative consequences on future educational and labour market success, lifetime earnings and later life health (e.g., Leopold, 2018; Tamborini et al., 2015; Oreopoulos, 2007).

However, thus far, no empirical evidence exists on how school closures and the COVID-19 pandemic affect the well-being of students in their final high school years, and how effects on their mental and physical conditions relate to their educational and career plans and transition outcomes. We fill this research gap by using large-scale panel data on well-being, educational and career plans and transition outcomes of 3697 German high school students we sampled from the 2020 and 2021 graduation cohorts. These data have two key features. First, they entail three detailed indicators of well-being, i.e., mental health (10-item Hopkins Symptom Checklist; Derogatis et al., 1974), self-rated physical health (5-point scale; e.g., Mossey & Shapiro, 1982), and life satisfaction (11-point scale; e.g., Lucas, 2007). Second, these data contain both pre-pandemic information and information during the pandemic, as they stem from three survey waves in fall 2019, spring 2020, and fall/winter 2020/21.

Drawing on these data enables us to investigate (i) the immediate effects of nationwide school closures on students’ well-being in spring 2020; (ii) the intermediate effects of the COVID-19 pandemic in general on students’ well-being in fall/winter 2020/21; (iii) the heterogeneous effects of the COVID-19 pandemic on high school graduates who transition to post-secondary education and students still enrolled in high school; and (iv) the impact of decline in mental health on career and educational plans and educational decisions of graduates.

In the first step of our analysis, we separately investigate immediate and intermediate effects of the COVID-19 pandemic on well-being. This separation is important, particularly with respect to school policies, since students may perceive school closures as holidays or health protection in the short run (Helliwell & Wang, 2014), while in the long run, stressors due to adverse health, learning achievement, distancing measures or uncertainty about the future may prevail. To evaluate the immediate effects of nationwide school closures, the data allow the application of a difference-and-differences design exploiting the fact that some students within the second survey wave responded just before and other students shortly after the school closures. To elaborate on the intermediate effects of the pandemic (i.e., the developments prior to and during the crisis), we employ linear growth curve modelling.

In a second step, we investigate whether the COVID-19 pandemic has different effects on students who spend most of their two final high school years in times of the pandemic (2021 graduation cohort) and students who graduated from high school shortly after the outbreak of the pandemic (2020 graduation cohort). Differences in well-being between graduation cohorts might occur because students still enrolled in high school may face greater uncertainty about future decisions than graduates who already realized their transition to post-secondary education. However, school graduates face a completely unknown situation at their new educational institutions, since universities and vocational schools have similarly introduced distance learning (Crawford et al., 2020), which hardly enabled any interactions with new fellow students and apprentices. Additionally, the pandemic has reduced the available vocational training positions as alternative educational paths after high school as well as the number of student jobs, which may affect the financial situations of university students (Yükselen et al., 2020). Thus, it remains open whether the pandemic and related distancing measures affect students still at school or school graduates differently.

In a final analysis step, this study investigates to what extent a severe decrease in mental health leads to changes in educational and career plans and transition outcomes. Investigating such associations is important because earlier work showed that pre-transition health positively influences university enrolment decisions (Zheng, 2017). Furthermore, poor mental health increases the probability of educational dropout (Cornaglia, Crivellaro, & McNally, 2015). One potential mechanism explaining the importance of mental health for educational decision making might be that, for instance, depressive symptoms alter perceptions of the future (e.g., Leykin et al., 2011; Roepke & Seligman, 2016). Thus, students with decreasing mental health may lose confidence in their educational and career plans or opt for transitions they would not have made with a better mental health status. As other major societal crisis, e.g., the Great Depression, have cumulative negative effects for individuals over the life course (Hale, 2017), investigating changes in mental health and educational plans and outcomes may provide important insights on the potential long-term consequences of the COVID-19 pandemic.

In analysing the immediate and intermediate effects of the COVID-19 pandemic on students’ well-being in two graduation cohorts and how changes in well-being relate to educational and career plans and transition outcomes, we extend the existing and rapidly emerging research on the effects of the COVID-19 pandemic on the well-being and mental health of young adults and teenagers (e.g., Elmer et al., 2020; Giuntella et al., 2021; Shanahan et al., 2020). Furthermore, we contribute to the literature on how lockdowns affect educational and career plans, which until now has concentrated on university students or employed individuals (Aucejo et al., 2020; Fiaschi & Tealdi, 2021). We combine these strands of literature and demonstrate that students who are close to the transition to post-secondary education are most vulnerable to shocks to their well-being due to the COVID-19 pandemic and that such shocks are related to educational and career plans and transition outcomes.

## Methods

### Participants and data collection

The participants of this study attended the highest track of secondary school in Germany, “*Gymnasium*”, in the final 2 years. The educational system comprises three tracks of secondary school: the lower and intermediate tracks prepare students for vocational training, whereas the highest (academic) track results—after successful completion—in the high school diploma *“Abitur”*, which qualifies students both to enrol at university or at a post-secondary vocational education or training. This academic track usually ends with final examinations at grade 12/13. These exams largely take place in March, and students receive their graduation diploma in the summer before they enrol at university or start vocational training in the fall.

After the outbreak of the COVID-19 pandemic at the beginning of 2020 and as one of the first nationwide pandemic prevention measures, all schools were closed after March 13th, 2020 in Germany. On April 23rd, 2020, the German federal states partly started reopening schools, albeit with very large regional and institutional variations: Since educational policy is the responsibility of the 16 federal states, there was no uniform school opening policy in Germany. Furthermore, local developments of the pandemic affected the closing of whole schools, class levels, or single classes. After the summer break, schools started on a regular basis and then went gradually back to limited schooling in November and December 2020, first by allowing only alternating groups of students from each classroom and then from January 2021 switching back to complete distance schooling.

**Fig. 1:**
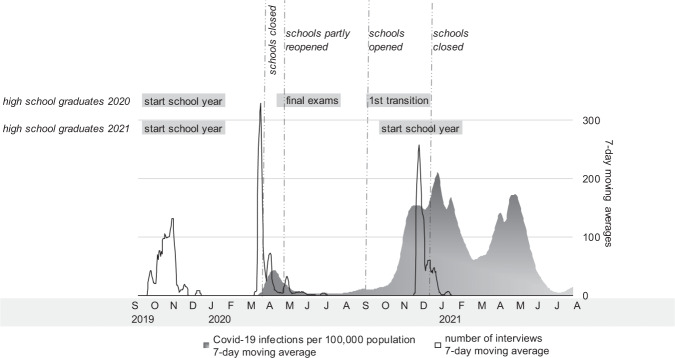
Timeline of data collection and COVID-19 infections in Germany

The data used in this study were collected for the BerO study, which evaluates the effectiveness of intensive job counselling for students in the highest secondary school track. The baseline survey (wave 1) was conducted as a paper-and-pencil interview (PAPI) in 214 schools in 8 of 16 German federal states. Students in these schools could voluntary complete the questionnaire in school between September and November 2019 and were instructed by a professional data collection team.^1^ In addition to these data, our analyses draw on data from two follow-ups, which took place outside the school context as a computer-assisted web or telephone interview (CAWI/CATI). Students were interviewed from February to June 2020 (wave 2) during the first wave of infection with some students answering before and others after school closures. Survey wave 3 took place from November to January 2021 during the second COVID-19 wave.^2^ Fig. 1 gives an overview of the timeline of the data collection.

### Measures

To answer the first and second research questions on the immediate and intermediate effects of the COVID-19 pandemic on the mental and physical well-being of young individuals, this study investigates changes over time in three outcomes: (i) mental health problems, (ii) life satisfaction, and (iii) self-rated health. The first three rows of Table 1 give an overview of the descriptive statistics of these outcome variables.

First, as a widespread measure for mental health, in waves 2 and 3, this study employs data from a subscale of the well-established Hopkins Symptom Checklist (HSCL-58; Derogatis et al., 1974) to approximate individuals’ risk for anxiety disorders and depression. The employed 10-item version (HSCL-10) has been shown to be a very good proxy for the longer HSCL-25 (Haavet et al., 2011; Schmalbach et al., 2021). This study uses a scale of four categories for each question (“No,” “Yes, a little,” “Yes, quite slightly,” and “Yes, extremely,” rated 1 to 4, respectively) and employs a binary measure, which indicates 1 if an individual’s average score on the 10-item scale exceeds the widely used cut-off point of 1.85. We used this binary predictor because trials have indicated clinically relevant symptoms of anxiety and depression above this cut-off point (Strand et al., 2003).^3^

Second, life satisfaction refers to “the degree to which an individual judges the overall quality of his/her own life as a whole favorably.” (Veenhoven, 2012: 66). For waves 1, 2 and 3, we employ the established 11-point scale (e.g., Lucas, 2007) and rely on answers to the following question: “How satisfied are you currently with your life in general?” Respondents could answer on a scale ranging from 0 (“totally dissatisfied”) to 10 (“totally satisfied”). Prior research on life satisfaction and mortality (e.g., Diener & Chan, 2011) indicates that such cognitive evaluations of individuals’ lives predict mortality.

Third, for waves 1, 2 and 3, this study examines self-rated health. Empirically, we rely on the question “How would you describe your current state of health?” Respondents could answer on a five-point likert-type scale ranging from 1 (“poor”) to 5 (“very well”). This question is a widely used item in many health studies in the social sciences and has been shown to be a strong predictor of mortality because it proxies general physical well-being (e.g., Mossey & Shapiro, 1982).

To elaborate on reasons for potential heterogeneity in well-being between graduation cohorts (i.e., between school students and school graduates), we additionally investigate whether students from the two cohorts perceive the COVID-19 situation differently. To this end, we explore whether differences in the current situation or whether worries about the future explain potential cohort variation. To approximate young individuals’ current situation, we use questions addressing enjoyment with learning and the extent to which young individuals are burdened by distancing measures. We approximate worries about the future in using questions asking about students’ worries about career plans (the descriptive statistics of these outcomes are shown in Table A1).

To answer the last research question, we identified severe drops in mental health between spring 2020 and fall/winter 2020/21 independent of the baseline value of mental health and the chosen cut-off point. A strong decrease is coded as 1 if values in the individuals’ HSCL-10 scores changed by at least 0.4 scale points. This applies to a quarter of the sample.

To investigate the influence of these severe mental health drops on educational and career plans, we rely on five different measures. For graduation cohort 2020 analysed educational and career plans comprise probabilistic beliefs about finishing the current post-secondary education. For graduation cohort 2021 analysed plans comprise probabilistic beliefs about successfully finishing potential university education, the certainty about the future educational pathway, expected final grade point average (GPA), and probabilistic beliefs of studying a science, technology, engineering and mathematics (STEM) major. While the probability of studying STEM directly measures one important educational aspiration, probabilistic beliefs and GPA expectations address—according to rational action theory (e.g., Breen & Goldthorpe, 1997; Cameron & Heckman, 1998)—important determinants of educational decisions.

To investigate the influence of decreasing mental health on educational transitions, we rely on three different outcomes after graduation, which were measured in the 2020 graduation cohort. Analysed measures comprise satisfaction with the overall educational decision, satisfaction with the new learning institution, and satisfaction with the location of this institution. Analysing satisfaction measures appears important because research shows that satisfaction outcomes are associated with educational dropout (e.g., Sarra, Fontanella, & Di Zio, 2019).

### Analytical strategy

#### Estimating the immediate effects of the first school closures

When investigating the effects of school closures, a pure outcome comparison between the students who answered before and after the school closures in March 2020 may be biased because the two groups of students may have different characteristics that could be related to our well-being outcomes under study. To solve this problem, we use the panel dimension of our data and apply a difference-in-differences estimation using data from waves 1 and 2 shown in Eq. 1:

1 $$Y_{it} = \beta _1SC_i + \beta _2W_{it} + \beta _3\left( {SC_iW_{it}} \right) + \varepsilon _{it}$$

where *Y*_*it*_ is the outcome of interest of individual i at wave t (life satisfaction and self-rated health, which are both available for waves 1 and 2). *SC*_*i*_ (**S**chool **C**losure) is a binary variable that takes a value of 1 for students who answered in March 2020 post school closure and 0 for students who answered in March 2020 pre school closure, and β_1_ captures the difference between those individuals. W_*it*_ (**W**ave) contains a wave dummy for Wave 2 interviews, where β_2_ captures the corresponding coefficient. ε_*it*_ is a standard error term. Finally, *SC*_*i*_W_*it*_ is the interaction term of *SC*_*i*_ and W_*it*_ that takes a value of 1 for students in wave 2 who answered the questionnaire post school closure. The coefficient β_3_ then measures the divergence in the outcome between those who answered post school closure, i.e., the treatment group, and those who answered pre school closure, i.e., the control group, which indicates the average treatment effect on the treated (ATT). This is the effect of the school closures.^4^

Only for the Hopkins scale we do not have information at wave 1. Therefore, for this outcome, we compare students who answered before and after the school closures, including a rich set of individual characteristics, as controls (shown in Table 1). For the analysis of all three outcomes, we restrict the time window to individuals who responded in the second survey wave prior to school closures and during closures. In doing so, the difference-in-differences analysis excludes students who participate in the interviews after school reopenings. Before the closures, all students answered within a time window of two weeks. Therefore, we argue that it is rather unlikely that pandemic factors, such as the infection rate, explain differences in well-being before and after school closures.

#### Estimating the development of well-being during the COVID-19 pandemic

To investigate the development of the examined well-being outcomes during the COVID-19 pandemic, we use the following specifications of linear random effects growth curve models^5^:

2 $$Y_{it} = \alpha + \beta _n\mathop {\sum}\limits_{n = 2}^3 {W_{nit}} + \lambda {^{\prime}}X_i + \mu {^{\prime}}X_{it} + \theta _i + \varepsilon _{it}$$

3 $$Y_{it} = \alpha + \beta _n\mathop {\sum }\limits_{n = 2}^3 W_{nit} + \gamma C_i + \delta _n\left( {C_i \times \mathop {\sum }\limits_{n = 2}^3 W_{nit}} \right) + \lambda {^{\prime}}X_i + \mu {^{\prime}}X_{it} + \theta _i + \varepsilon _{it}$$

In both equations, *Y*_*it*_ represents either life satisfaction (0 to 10), self-rated health (1 to 5) or mental health problems, which are approximated by the risk for anxiety disorders and depression (0 vs. 1). *θ*_*i*_ represents a person-specific error term, which is modelled as a random variable. *ε*_*it*_ constitutes an idiosyncratic error term. *W*_*nit*_ indicates dummy variables for each survey wave. *X*_*it*_ indicates a vector with time-varying confounding variables, whereas *X*_*i*_ captures time-constant confounders. In Eq. 3, we introduce *C*_*i*_ indicating whether a respondent stems from the 2021 or 2020 graduation cohort. To allow for variation across graduation cohorts, we interact *C*_*i*_ with each wave dummy. While the multiplicative effect of *γ* captures heterogeneity between cohorts at wave 1, *δ*_*n*_ captures heterogeneity in well-being between cohorts over the course of the COVID-19 pandemic.

Finally, we apply two sets of ordinary least square regressions. In the first set, we specify a model to elaborate on differences between graduation cohorts 2020 and 2021 at wave 3 (fall/winter 2020/21). In the second set, we identify individuals with strong decreases in mental health between survey waves 2 and 3 to generate a binary variable (reference group: slight or no decrease in mental health) and regress educational and career plans and transition outcomes at wave 3 on this binary indicator.^6^ In correlating these measures, we elaborate on the potential long-term impact of the COVID-19 pandemic. Although this procedure constitutes a correlative workaround due to potential reversed causality, we can rule out parts of endogenous selection bias by using our rich data. To this end, our model specifications condition on a vast set of individual characteristics as control variables, described in “Sample characteristics and control variables”, and they also include the baseline level (i.e., survey responses given in fall 2019 that are independent of any COVID-19-related factors) of each dependent variable and the baseline level of mental health (measured at wave 2).

### Sample characteristics and control variables

From the BerO baseline sample (*N* = 7192), we construct a balanced sample and restrict our analyses to students who participated in all three waves (fall 2019, spring 2020 and fall/winter 2020/21) with nonmissing information on our outcomes or metric control variables. If information is missing in a categorical control variable, we create a missing information category. As one main question of this study is as to what extent mental well-being declines correlate with perceived post-secondary transition outcomes, we further exclude individuals from the analytical sample that transitioned from high school to a “gap year” or standby state. In this year young individuals typically bridge the time between high school graduation and enrolling at university or starting vocational training. Since such a sample truncation has the potential to distort our main findings (e.g., Elwert and Winship, 2014), we re-run all analyses on a full sample. This robustness check reveals that our main results also hold if we include graduates in a “gap year” (Figures A2 to A4 in the Online Appendix). Overall, our balanced sample consists of 3697 students. 2451 students stem from graduation cohort 2021 and 1246 students from graduation cohort 2020.

The set of individual characteristics that we use as controls includes socio-demographics, i.e., cohort (graduation cohort 2021 dummy), gender (male dummy), migration background (1st/2nd generation migrant), parental education (at least one parent with university education), and educational achievement (GPA better than 2.5 on average on a scale from 1 – best grade to 6 – failed). For these characteristics, the strong gender imbalance is notable. This imbalance is partly because women are in general overrepresented in German high schools and because women tend to participate in questionnaires more often (e.g., Becker, 2021). Moreover, we use a rich set of preferences, i.e., risk aversion and myopia, and personality traits, for which we use constructs based on multiple items to measure self-efficacy, grit, and the Big Five personality dimensions openness, conscientiousness, extraversion, agreeableness, and neuroticism. We also include a dummy variable for interview mode to account for possible mode effects and, finally, rely on school fixed effects to take into account institutional and geographical variation in the data.

Table 1 presents the sample characteristics in the three waves, with the spring 2020 wave split into individuals who answered before and after the school closures. The first rows show the means of our overall well-being measures. They show strong variation among the waves and before and after the school closures. We will investigate these changes in detail in the next sections. The next rows depict the sociodemographic characteristics, educational achievement and educational choice, as well as preferences and personality traits of the sample. As we use a balanced sample, there are no differences in these characteristics between wave 1 (fall 2019) and wave 3 (fall/winter 2020/21). However, the figures reveal that the characteristics between those students who answered before and after the school closures differ; for example, more males and slightly worse performing students answered the questionnaire after the school closures compared to students who participated in the survey before the school closures. This finding supports our strategy to apply a difference-in-differences approach to rule out biases by this selection.

Appendix Table A1 shows the descriptives of the measures, which are only available for wave 3 (fall/winter 2020/21). The variables contain information on attitudes and worries, i.e., how students deal with the COVID-19 pandemic and distancing measures, as well as on educational and career plans and further sociodemographic characteristics. Appendix Table A2 shows the aggregated values over the three waves for the variables shown in Table 1.

**Table 1:** Sample characteristics by wave

Means	Fall 2019	Spring 2020		Fall/Winter 2020/21
	Oct. to Nov.	Pre-SC	Post-SC	Nov. to Jan.
Outcomes				
Life satisfaction (0–10)	7.435	7.080	7.170	6.671
	(1.940)	(1.993)	(2.001)	(2.107)
Self-rated health (1–5)	3.872	3.634	3.891	3.684
	(1.022)	(1.081)	(0.998)	(1.103)
Mental health problems (1 vs. 0)	–	0.425	0.334	0.485
		(0.494)	(0.472)	(0.500)
Socio-demographics				
Graduation cohort 2021 (1 vs. 0)	0.663	0.653	0.678	0.663
Male (1 vs. 0)	0.355	0.330	0.395	0.355
1st/2nd generation migrants (1 vs. 0)	0.209	0.206	0.214	0.209
Missing information on migration status (1 vs. 0)	0.055	0.058	0.050	0.055
At least one parent with university degree (1 vs. 0)	0.540	0.526	0.562	0.540
Missing information on parental education (1 vs. 0)	0.105	0.105	0.105	0.105
GPA better than 2.5 (1 vs. 0)	0.484	0.495	0.465	0.484
Missing information on GPA (1 vs. 0)	0.010	0.008	0.013	0.010
Attending university (1 vs. 0)	–	–	–	0.284
Attending vocational training (1 vs. 0)	–	–	–	0.054
Preferences and personality				
Risk aversion (0–10)	5.629	5.567	5.730	5.629
	(2.183)	(2.187)	(2.173)	(2.183)
Self-efficacy (1–4)	2.924	2.920	2.931	2.924
	(0.407)	(0.412)	(0.398)	(0.407)
Grit (1–5)	3.472	3.488	3.446	3.472
	(0.614)	(0.619)	(0.606)	(0.615)
Big Five				
Openness (1–7)	4.778	4.764	4.802	4.778
	(1.220)	(1.233)	(1.199)	(1.220)
Conscientiousness (1–7)	5.222	5.258	5.165	5.222
	(1.022)	(1.020)	(1.025)	(1.023)
Extraversion (1–7)	4.764	4.707	4.857	4.764
	(1.381)	(1.395)	(1.354)	(1.382)
Agreeableness (1–7)	5.434	5.421	5.454	5.434
	(0.947)	(0.953)	(0.937)	(0.947)
Neuroticism (1–7)	4.245	4.259	4.221	4.245
	(1.234)	(1.243)	(1.221)	(1.234)
Dummy for being myopic (1 vs. 0)	0.115	0.114	0.117	0.115
Interview method				
CATI interview (1 vs. 0)	–	–	0.053	0.052
PAPI/CAWI interview (1 vs. 0)	1.000	1.000	0.947	0.948
*N* persons	3697	2292	1405	3697

*Notes:* Standard deviations in parentheses. *GPA* Grade point average, a lower GPA indicates better performance, *CATI* Computer-assisted telephone interview, *PAPI* Paper-and-pencil interview, *CAWI* Computer-assisted web interview, *SC* School Closures. Mental health problems equals 1 if an individual’s average score on the HSCL-10 item scale exceeds the cut-off point of 1.85

Data: BerO study wave 1, 2 and 3

## Results

### Immediate effects of first school closures on well-being

Table 2 presents the immediate effects of school closures on the three well-being outcomes. Using the difference-in-differences approach explained in Eq. 1 demonstrates that while school closures did not affect life satisfaction, self-rated health weakly increased after school closures by 0.21 standard deviations (see online Appendix Figure A1 for a graphical illustration of the effects). Investigating the immediate effect of school closure on mental health based on a within-wave 2-comparison reveals that the risk of mental health problems—while controlling for the variables introduced in “Sample characteristics and control variables”—is 5 percentage points lower for the students who participated in the survey after the closures. Overall, these results indicate that school closures had a positive effect on overall health in the first weeks after their implementation, as indicated by improvements in two of the three measures.

**Table 2:** Immediate effects of school closures on well-being: Results from difference-in-differences and OLS regressions

	Mean wave 1 Pre SC Group	Mean wave 1 Post SC Group	Mean wave 2 Pre SC Group	Mean wave 2 Post SC Group	DID in % of SE	p-value DID
Panel A						
Life satisfaction (0–10)	7.392	7.509	7.080	7.187	−0.005	0.872
Self-rated health (1–5)	3.863	3.899	3.634	3.882	0.213	0.000
Panel B					Mean Diff. pre and post school closures	*p*-value
Mental health problems (0–1)			0.434	0.354	−0.053	0.000
*N* persons	2292	1211	2292	1211		

*Notes:* Panel A presents estimates in percent of standard deviation based on difference-in-difference regressions with federal state fixed effects. Panel B presents mean differences based on an OLS regression. Controls: school fixed effects, gender, migration status, parental education, school performance at wave 1, self-efficacy, Grit, Big Five personality traits, graduation cohort, risk aversion, time preferences, self-rated health, life satisfaction and interview mode. *SC* School Closures

Data: BerO study wave 1 and 2

To test whether our treatment indicator actually picks up the effect of school closures, we ran a placebo test. In doing so, we assigned all individuals who answered in survey wave 2 in calendar week eleven (one week before the school closures) and onwards to the post school closure group. As a consequence the post school closure comprises a large amount of respondents (around 1700) who were not affected by school closures. The results from this robustness check suggest that the increase in self-rated health and the decrease in mental health problems are substantially smaller and statistically non-significant. Thus, our main specification appears to pick up the immediate effects of nationwide school closures.

### Development of well-being before and during the first and second waves of the COVID-19 pandemic

**Fig. 2:**
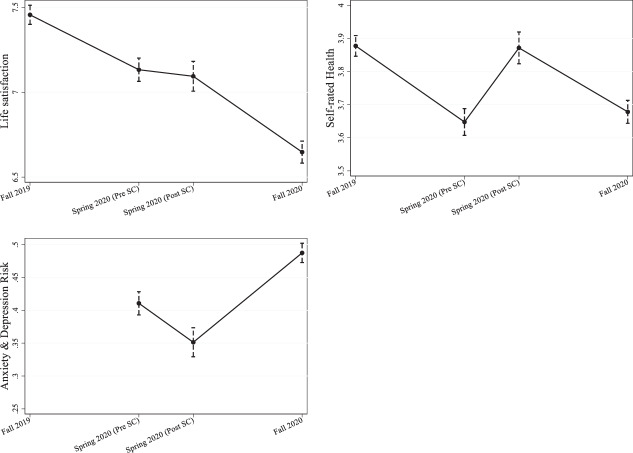
Development of well-being since fall 2019. Results from random effect growth curve models. *Notes:* Outcomes: Life satisfaction (0 to 10); self-rated health (1 to 5); Dummy for being above the clinical threshold for a high anxiety and depression risk (HSCL-10). N Life Satisfaction = 11,091; N SRH = 11,091; N HSCL-10 = 7394. Controls: school fixed effects, gender, migration status, parental education, school performance at wave 1, self-efficacy, Grit, Big Five personality traits, graduation cohort, risk aversion, time preferences, and interview mode. Data: BerO study wave 1 to 3

In this section, we investigate the effects of school closures and distancing regulations eight months after the pandemic started. Figure 2 shows the development of the three outcomes at wave 1, at wave 2 before and after the school closures and at wave 3 calculated by applying Eq. 2 (see Appendix Table A3 for point estimates and significance levels). In line with the previous section, we see an immediate increase in self-rated health and a decrease in mental health problems in wave 2 after the school closures. However, from spring to fall, we observe a strong decrease in life satisfaction and self-rated health and a particularly strong increase in mental health problems. Overall, the data suggest that after students’ overall health improved in the short term, it strongly declined in the longer term.

### COVID-19 effects on mental and physical well-being by graduation cohorts 2020 and 2021

**Fig. 3:**
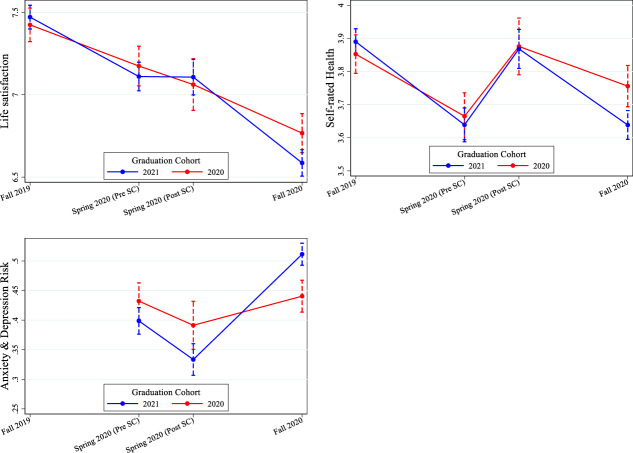
Development of mental and physical well-being by graduation cohort. Results from random effect growth curve models. *Notes:* Outcomes: Life satisfaction (0 to 10); self-rated health (1 to 5); Dummy for being above the clinical threshold for a high anxiety and depression risk (HSCL-10). N Life Satisfaction = 11,091; N SRH = 11,091; N HSCL = 7394. Controls: school fixed effects, gender, migration status, parental education, school performance at wave 1, self-efficacy, Grit, Big Five personality traits, risk aversion, time preferences, and interview mode. Data: BerO study waves 1 to 3

We now investigate how the effects on the three well-being measures differ over time between the 2020 and 2021 graduation cohorts. Using Eq. 3, Fig. 3 shows that none of the three measures differed significantly at wave 1 or 2 (before and after the school closures) between the two cohorts (see Appendix Table A4 for the point estimates and significance levels). However, at wave 3 (fall/winter 2020/21), the graduation cohort 2021 (graduating in spring 2021) showed significantly worse outcomes for all three well-being measures. The difference was most pronounced for mental health problems, for which the increase for graduation cohort 2021 from wave 2 to wave 3 amounts to almost 20 pp, while the increase was 5 pp for the 2020 graduation cohort.

After showing substantial variation between graduation cohorts in fall/winter 2020/21, we now investigate two potential mechanisms that might explain cohort differences. In particular, we test whether differences in the current enjoyment of education and burdens induced by distancing measures or whether worries about the future can explain cohort variation. We assume that the mechanisms under study are important because young individuals spend a great amount of their daily time with being at school and with school work (Anger et al., 2020). Moreover, research indicates that perceived career insecurity is detrimental to well-being (e.g., Kopasker, Montagna, & Bender, 2019).

For this purpose, Columns 1 and 2 of Table 3 present the results of two sets of questions that the students in both cohorts answered at wave 3, and Column 3 shows the differences between the two graduation cohorts. Students who had just started their final school year (graduation cohort 2021) reported higher levels of burden due to distancing measures and less enjoyment of learning in fall 2020 than those who already had left school (graduation cohort 2020) and had already attended university study (84%) or vocational training (16%). Analysing respondents’ worries about their future reveals that students from graduation cohort 2021 were more worried than those in graduation cohort 2020 about their occupational futures and expected negative effects of distancing policies on their future careers. They also claim deficits with respect to receiving relevant career information.

**Table 3:** Attitudes of the 2020 and 2021 graduation cohorts regarding distancing measures and worries (Fall 2020)

	(1) Mean graduation cohort 2021	(2) Mean graduation cohort 2020	(3) Diff. btw. Cohorts 2021–2020
Dealing with the current situation			
Enjoyment of learning (1–5)	3.052 (*1.052*)	3.715 (*0.958*)	−0.662***
Burden of distancing measures (1–5)	3.036 (*1.304*)	2.624 (*1.228*)	0.412***
Future worries			
Impact of distancing policies on future career (0 vs.1)	0.622 (*0.484*)	0.447 (*0.497*)	0.175***
Worries about occupational future (1–5)	2.322 (*1.191*)	1.959 (*1.150*)	0.363***
Worries about too little career information (1–5)	3.058 (*1.337*)	2.500 (*1.359*)	0.558***
*N* persons	2450	1247	

*Notes:* Standard deviations italicized in brackets

*p  <  0.10, **p  <  0.05, ***p  < 0.01 indicate significant differences (based on *t*-tests)

Data: BerO study wave 3

Next, we investigate to what extent these higher concerns of the graduation cohort 2021 can explain the observed differences in well-being between the two cohorts. Table 4 shows the results of the estimations in which we regress the three well-being measures on the control variables (column 1) and on the two sets of questions that may explain the difference between the cohorts in well-being (Columns 2 and 3). In line with the results in Fig. 3, Column 1 demonstrates that graduation cohort 2021 reported significantly worse outcomes in the health measures than graduation cohort 2020. However, the results in column 2 show that including the present attitudes completely absorbs the difference between the cohorts for all outcomes and explains the largest share of the difference in life satisfaction, self-rated health, and mental health between the cohorts. The present attitudes explain even more of the gap than future worries, which also reduces the effect of the graduation cohort but does not fully absorb it (column 3).

**Table 4:** Mechanisms explaining differences in dimensions of well-being between graduation cohorts 2020 and 2021

	(1)	(2)	(3)	(4)
	Baseline model	+ current situation	+ future worries	+ current situation & future worries
Panel A: Mental Health Problems^*b*^				
Graduation cohort 2021 dummy	0.095***	0.025	0.059***	0.003
Enjoyment of current education		−0.090***		−0.084***
Burden of distancing measures		0.023***		0.016**
Impact of distancing policies on future career			0.044***	0.029*
Worries about occupational future			0.040***	0.032***
Worries about too little career information			0.019***	0.018***
R^2^	0.233	0.267	0.251	0.278
Panel B: Self-rated Health^a^				
Graduation cohort 2021 dummy	−0.058***	0.005	−0.028	0.023
Enjoyment of current education		0.187***		0.178***
Burden of distancing measures		−0.051***		−0.033*
Impact of distancing policies on future career			−0.056***	−0.043**
Worries about occupational future			−0.065***	−0.047**
Worries about too little career information			−0.046**	−0.043**
R^2^	0.147	0.178	0.160	0.186
Panel C: Life Satisfaction^a^				
Graduation cohort 2021 dummy	−0.067***	0.016	−0.038*	0.034*
Enjoyment of current education		0.273***		0.263***
Burden of distancing measures		−0.018		−0.000
Impact of distancing policies on future career			−0.045***	−0.036**
Worries about occupational future			−0.090***	−0.065***
Worries about too little career information			−0.030*	−0.027
R^2^	0.183	0.242	0.197	0.250
*N* persons	3697	3697	3697	3697

*Notes:* ^a^Standardized beta coefficients from OLS regressions

^b^Predicted probabilities from OLS regressions

Statistical significance at: *p  <  0.10, **p  < 0.05, ***p  < 0.01

Dependent variables: Life satisfaction (0–10), self-rated health (1–5), and Dummy for being above the clinical threshold for mental health problems (HSCL-10)

Control variables: school fixed effects, gender, migration status, parental education, school performance at wave 1, self-efficacy, Grit, Big Five personality traits, risk aversion, time preferences, interview mode, subjective household income, and a dummy for an unemployed relative

Data: BerO study wave 3

### Associations between mental health decreases and educational and career plans and transition outcomes

In this section, we analyse whether and to what extent the decrease in mental health that we observe from spring 2020 to fall/winter 2020/21 is related to students’ educational and career plans and transition outcomes. We focus on mental health, for which we find the strongest decrease between spring 2020 and fall/winter 2020/21. As described in “Analytical Strategy”, we regress educational and career plans and transition outcomes, measured in fall/winter 2020/21, on a dummy that takes a value of 1 if a student showed a strong decline in mental health (i.e., an increase on the HSCL-10 scale above a value of 0.4, which represents the upper quartile) from spring to fall/winter. The first model controls for student characteristics, while the second model additionally uses the panel dimension of our data and includes mental health values at wave 2 (i.e., the first measure of mental health available) and the dependent variable of the model, i.e., the educational and career plans at wave 1 (i.e., the first measure that is independent of the COVID-19 situation) as control variables. Models investigating transition outcomes include life satisfaction at wave 1 as a control. These two additional controls exclude the possibility that our estimates merely capture the effect of a student’s generally low mental health and that those students with a decrease in mental health would have already stated less ambitious educational and career plans and dissatisfaction before the decrease.

Table 5 shows the results of the estimations for the 2020 graduation cohort (Columns 1 and 2) and for the 2021 graduation cohort (Columns 3 and 4). In both cohorts, a strong decline in mental health was related to a lower success probability of the current educational path or potential future university education. The next rows present the results for educational and career plans among graduation cohort 2021 and transition outcomes among graduation cohort 2020. For the 2020 graduation cohort, the results reveal that students with a strong decline in mental health were less satisfied with their overall current educational decision, location, and institution than students with a lower or no decline. In line with these results, students with a strong decline in mental health in the 2021 graduation cohort stated that they felt less secure about their future career paths, they expected a worse GPA, and they had a lower probability of wanting to study STEM subjects. Columns 2 and 4 demonstrate that these results hold in the very tight specification, which includes lagged mental health and lagged educational and career plans and satisfaction. The sizes of the effects (between 10 and 15% of a standard deviation) are higher for the 2020 graduation cohort than for the 2021 cohort. However, for the 2021 cohort, the effects were in a relevant range, with approximately five percent of a standard deviation.

**Table 5:** Associations between strong decreases in mental health and educational and career plans and transition outcomes in fall/winter 2020/21

	Cohort 2020		Cohort 2021	
Outcomes in fall/winter2020/21	(1) Base model	(2) + Baseline value of DV & HSCL-10	(3) Base model	(4) + Baseline value of DV & HSCL-10
Success probability^a^				
Strong HSCL-10 increase	−0.093**	−0.118***	−0.064**	−0.067***
R^2^	0.269	0.308	0.172	0.271
*N* persons	1244	1244	2447	2447
Security of educational path				
Strong HSCL-10 increase	–	–	−0.038*	−0.043**
R^2^	–	–	0.155	0.254
N persons	–	–	2449	2449
*Expected GPA*				
Strong HSCL-10 increase	–	–	0.050**	0.044**
R^2^	–	–	0.523	0.674
N persons	–	–	2372	2372
Probability of STEM Studies				
Strong HSCL-10 increase	–	–	−0.035*	−0.020
R^2^	–	–	0.286	0.684
N persons	–	–	2414	2414
Satisfaction with decision^b^				
Strong HSCL-10 increase	−0.133***	−0.158***	–	–
R^2^	0.275	0.305	–	–
N persons	1242	1242	–	–
Satisfaction with location^b^				
Strong HSCL-10 increase	−0.081**	−0.089**	–	–
R^2^	0.258	0.268	–	–
N persons	1177	1177	–	–
Satisfaction with institution^b^				
Strong HSCL-10 increase	−0.124***	−0.141***	–	–
R^2^	0.256	0.282	–	–
N persons	1181	1181	–	–

*Notes:* Standardized beta coefficients. Statistical significance at *p <  0.10, **p  <   0.05, ***p < 0.01. *GPA* Grade point average, a lower GPA indicates better performance, *HSCL*-*10* Hopkins Symptom Checklist, 10-item version

Dependent variables: Success probability (11-point Likert scale), security of educational path (5-point Likert scale), probability of STEM Studies (11-point Likert scale), satisfaction measures (11-point Likert scale)

Control Variables: graduation cohort (Panel A), gender, migration status, school performance, school fixed effects, parental education, subjective household income, parental unemployment in last 6 months, onsite education, self-efficacy, grit, time preference, risk aversion, Big Five personality traits, and interview mode

Overall, 26.81% of the sample experienced a strong decrease in mental health (i.e., an increase on the HSCL-10 scale of more than 0.4 points). The share of strong decreases was stronger among the 2021 graduation cohort, in which 29.92% of respondents exhibited a strong decline in mental health, while the corresponding share from the 2020 graduation cohort was only 20.69%

^a^Success probability for the 2020 cohort refers to the likelihood of finishing the current post-secondary education. For the 2021 cohort, success probability refers to the likelihood of successfully finishing a potential future academic study

^b^The baseline value here constitutes overall happiness at survey wave 1

Data: BerO study wave 3

## Discussion

We start the discussion of the results with the question of why school closures create positive effects on well-being in the short run. Our first explanation for the positive short-run effects refers to the idea that students perceived school closures as a relief, hence resembling additional holidays. This explanation is based on the finding that individuals show higher well-being on weekends and during holidays (e.g., Ryan et al., 2010), which in the case of students may be caused by the fact that studying provides less well-being than other leisure activities (Helliwell & Wang, 2014). Additional studies have shown that if high school students are not at school, they feel less stressed because of reduced pressure and bullying, which in extreme cases even leads to less suicide during the holiday months (Hansen & Lang, 2011, Kim & Leventhal, 2008). The finding that students spend much fewer hours studying during school closures than in normal times supports the holiday explanation (Anger et al., 2020 for the present sample; Grewenig et al., 2020, Grätz & Lipps, 2021).

An alternative explanation for the increase in well-being might be that students perceive school closures as a measure to protect their health and relieve their fear of becoming infected with COVID-19. The finding that a positive effect exists for self-rated health and mental health problems while the effect is absent for life satisfaction may support this explanation. In addition, the decline in self-rated health and life satisfaction from Wave 1 to Wave 2 for the students who answered the questionnaire before the school closures supports the health protection explanation, as the decline may have resulted from COVID-19 fear. Finally, self-rated health for students who answered the questionnaire after the school closures remained at the same level as in wave 1 and did not increase. In the case of a holiday effect, we may have expected an increase in this outcome. However, interview mode or honesty-in-reporting effects may also explain the decline in life satisfaction and self-rated health from wave 1 to wave 2, which supports the holiday explanation (Chadi, 2013; Warren & Halpern-Manners, 2012). Irrespective of the final explanation for the increase in well-being shortly after school closures, the findings are policy relevant, as they demonstrate that short-term school closures are not harmful to students’ well-being.

Next, we discuss why well-being declines in the long run and why this decline is stronger for students who are still enrolled at high schools. In the longer run, the burdens of school closures and other distancing measures may accumulate over time because students suffer more from social distancing and home schooling and may be afraid about a loss of human capital. Additionally, students increasingly perceive the pandemic not only as a short-term event but also as a long-term condition. This is an important finding, because our results implicate that physical and mental well-being developed very dynamically during the pandemic and measuring well-being at one point in time during a crisis may be misleading. Svaleryd & Vlachos, (2022) describe positive and negative effects on mental health and well-being in their literature review. For example, Sachser et al., (2021) also found positive immediate effects of lockdown measures on mental health in a representative sample of the German population without assessing longer-term outcomes. In contrast, there are studies which also found short-term decreases in well-being, particularly for mothers and working parents in general (e.g., Cheng et al., 2021; Huebener et al., 2021). Taken together, the results suggest the importance of differentiating by mechanisms, groups, and time points when researching mental health and well-being.

As an explanation for the observed long-run decline in well-being of the 2021 graduation cohort, we find that students who were still in school were more worried about their future careers and were more burdened by the current COVID measures, in particular school closures, social distancing measures during phases of reopened schools and perceived future career uncertainty. These differences in perceptions explain the difference in decline in overall well-being almost completely.

The existence and explanation of the difference between the cohorts are surprising, as the high school graduates from the 2020 cohort were also strongly affected by distancing measures, such as having online lectures or prohibitions of freshman events, and uncertainties about the future. However, in the first post-high school year, individuals from graduation cohort 2020 appeared to cope better with the situation. One explanation for the finding may be that freshmen were not familiar with universities, vocational schools or training firms without distancing measures and that therefore, they did not miss anything, for example, on-site lectures. Additionally, after having made a successful transition, worries about the future may have decreased. In contrast, high school students still have to make the transition out of school and are therefore exposed to greater uncertainty. Overall, the results indicate that students who were locked down while still in school are most vulnerable to an overall health decline, which should be considered in prevention or support services.

Finally, it is important that the decline in well-being is related to educational and career plans and satisfaction with the chosen educational path. As students from the 2020 graduation cohort with a decline in mental health reported less satisfaction with their choices, they were more likely to drop out, causing high costs for the individual and society. The same is true for the 2021 graduation cohort: our results, i.e., that these students feel less secure about the future, suggest that they will make decisions that they would have not made without the pandemic, which may also lead to high individual and societal costs.

Although our study has many strengths, it also shows limitations. First, we cannot state whether the COVID-19 distancing measures causally generated the decline in physical and mental well-being, as we do not have a control group who was not affected by the pandemic policies. However, it is very unlikely that only time, seasonal effects or any other event caused the reduction, as the decline was too strong for these explanations. Similar declines only occur in regions where a war started or in individuals who have experienced a stroke of fate, such as becoming widowed or disabled (Coupe & Obrizan, 2016; Infurna et al., 2017; Oswald & Powdthavee, 2008). Furthermore, other studies showed that well-being remained stable during the final years of high school education for cohorts who graduated before the pandemic (Herke et al., 2019). A second limitation might be that the size of the relationship between the decline in mental health and the transition outcomes in our estimates is not very large. Nevertheless, the relationships are meaningful because a rich body of research based on the notion of cumulative (dis)advantage shows that even small changes in this critical stage of the life course can have long-lasting accumulating effects over the life course (e.g., DiPrete & Eirich, 2006).

## Conclusion

This paper analyses how the COVID-19 pandemic and the related measures to reduce the spread of the coronavirus have affected the well-being, educational and career plans and transition outcomes of students from the 2020 and 2021 high school graduation cohorts. The results show that after an immediate increase in physical and mental health around the time of the first school closures, well-being strongly declined in the longer run, particularly for students in the 2021 graduation cohort, who were still in school at the time of the survey in fall/winter 2020/21. Additionally, our results demonstrate that this decline in well-being was related to several educational and career plans and transition outcomes. The results clearly indicate that the COVID-19 pandemic, including school closures and distancing measures, has had negative effects on current graduation cohorts, which may cause life-long harm. Next, disentangling the effects of different pandemic policies, e.g., school closures or shutdown of leisure activities, would be crucial to evaluate the true costs and benefits of such policies. This is particularly important because the effectiveness of school closures as one of the main policies to prevent COVID-19 infections is disputed (Courtemanche et al., 2021; Isphording et al., 2021; van Bismarck-Osten et al., 2021). Finally, we address the question of intergenerational equality, as our findings demonstrate that the younger generation is likely to bear the long-term costs of pandemic policies, while the benefits of distancing measures in terms of lower infections are likely to be higher for older individuals.

## Supplementary Information

Sandner_etal_COVID_Wellbeing_HighSchoolStudents_Appendix_

## Footnotes

1 The voluntary participation in the first wave within schools and in the follow-up waves outside the school context means that the survey data is not necessarily representative for the whole population of German high school students in their final years. However, we compare some of the outcome measures (self-rated health, life satisfaction) of our sample with data from the representative SOEP Youth Survey and find similar pre-pandemic values. Furthermore, to account for different response rates and any other characteristics which may differ between schools, we include school fixed effects.

2 To investigate whether attrition between the panel waves may be selective, we analyzed the determinants of sample inclusion in the balanced panel. In particular, individuals from the younger graduation cohort (cohort 2021) were more likely to stay in the sample, which can be explained by the higher mobility of the older graduation cohort (cohort 2020) after their high school degree. While there seems a slightly positive selection of individuals in the balanced panel (e.g., high-performing students), there is no indication that our outcome variables determine the participation in all three waves.

3 As a robustness check, we also conducted the analysis using the original variable for mental health to allow for more variation in the outcome variable, which confirmed the results of our main analysis.

4 As those individuals who were exposed to the school closures also have experienced a significantly longer exposure to the pandemic in general, our school closure effects may also capture effects of the pandemic in general. To address this threat, in Table A6 in the Appendix we show that intra-individual differences in interview timing between wave 2 and 3 after the outbreak of the COVID-19 pandemic (see Figure A5 for an overview of differences in response timing), which we use as a proxy for pandemic exposure, has no substantial effect on well-being outcomes under study. In respect to our Diff. in Diff. approach, the results presented in Table A6 suggest that the positive short-term school closure effects on wellbeing outcomes are a lower bound as the time effect points in another direction and leads to a decline in well-being (although mainly not statistically significant and very small in size). As the employed fixed-effects approach uses a subsample of respondents who answered in survey wave 2 after school closures and in survey wave 3 (N=1,405) and relies on strong exogeneity assumptions, we want to stress that these results constitute rather stylized findings on pandemic exposure on our well-being outcomes. Nevertheless, they strengthen our results retrieved from Eq. 1.

5 We use linear random effects growth curve models mainly because these models allow for individual-specific changes in the outcomes under study over time (i.e., they incorporate an individual- and time-specific random effect). To this end, the random effect model uses individual-specific and between-person variation thereby allowing to investigate how group differences emerge over time, a special feature of the model we heavily rely on in our study. The use of within-person variation constitutes a major advantage compared to pooled OLS. The advantage over FE models constitutes the fact that RE growth curve models allow investigating group differences (of course at the cost of potential bias due to individual-specific time constant unobserved heterogeneity). In Table A7 in the appendix we show, however, that results from RE, OLS and FE models do hardly differ.

6 We show results of linear probability models, as these are more intuitive and easy to interpret. However, to take into account that using a non-linear binary choice estimator may be more accurate for binary outcome variables, we also conduct analyses using an ordered logit model for life satisfaction and self-rated health, as well as a logit model for mental health. The coefficients of these non-linear models are very similar to those revealed by our main analyses.
