# Supplementary material for: The COVID-19 pandemic, well-being, and transitions to post-secondary education
Source: Rev Econ Househ. 2022 Sep 23;21(2):461–83. doi: 10.1007/s11150-022-09623-9 (PMC9510585; doi:10.1007/s11150-022-09623-9)
Supplement: Supplementary file 1 — Sandner_etal_COVID_Wellbeing_HighSchoolStudents_Appendix_ [file 11150_2022_9623_MOESM1_ESM.pdf]

Online Appendix

Table A1 Variables only included in fall/winter 2020/21

	Mean	Std. Dev.	Min	Max
**Dealing with the current situation**				
Fun with current education	3.276	1.068	1	5
Burden distancing measures	2.897	1.294	1	5
Worries about occupational future	2.200	1.190	1	5
Worries about too less career information	2.870	1.370	1	5
Distancing policies have impact on future career	0.563		0	1
**Educational plans**				
Security educational path	3.666	1.134	1	5
Success probability of finishing pot. study	8.258	1.803	0	10
Success probability of finishing current post-sec. edu.	7.742	2.272	0	10
Satisfaction with decision	7.407	2.055	0	10
Satisfaction with location	7.381	2.234	0	10
Satisfaction with institution	7.500	1.915	0	10
**Strong increase in anxiety & depression risk**	0.268		0	1
**Control variables for wave 3-specific analyses**				
Dummy for an unemployed relative	0.257		0	1
Subjective household income				
1 much less than one needs for a decent life	*0.012*			
2	*0.062*			
3	*0.229*			
4	*0.549*			
5 much more than one needs for a decent life	*0.140*			
Missing information on subj. HH income	0.009			
Onsite education at time of interview	0.641			
Baseline fun with education	3.300	0.890	1	5
Baseline security with educational path	3.545	1.135	1	5
Baseline success probability of finishing pot. study	8.517	1.573	0	10
Baseline happiness	7.435	1.940	0	10
N persons	3,697			

Table A2 Overall Sample characteristics

	Mean	Std. Dev.	Min	Max
**Outcomes**				
Life satisfaction	7.074	2.040	0	10
Self-rated health	3.763	1.065	1	5
Anxiety & depression risk	0.438	0.496	0	1
**Socio-demographics**				
Graduation cohort 2021	0.663		0	1
Male	0.355		0	1
1st/2nd generation migrants	0.209		0	1
Missing information on migration status	0.055			
At least one parent with university education	0.540		0	1
Missing information on parental education	0.105			
GPA better than 2.5	0.484		0	1
Missing information on GPA	0.010			
**Personality and Preferences**				
Self-efficacy	2.924	0.407	1	4
Grit	3.472	0.615	1	5
Dummy for being myopic	0.115		0	1
Openness	4.778	1.220	1	7
Conscientiousness	5.222	1.023	1.333	7
Extraversion	4.764	1.381	1	7
Agreeableness	5.434	0.947	1	7
Neuroticism	4.245	1.234	1	7
Risk aversion	5.629	2.183	0	10
**Method**				
CATI interview	0.024		0	1
Observations	11,091			
N persons	3,697			

Table A3 Development of mental and physical health since fall 2019. Results from random effect growth curve models

	Life-satisfaction	Self-rated Health	Anxiety & Depression risk
Spring 2020 (Pre SC)	-0.323***	-0.230***	
	(0.036)	(0.022)	
Spring 2020 (Post SC)	-0.362***	-0.006	-0.059***
	(0.045)	(0.026)	(0.014)
Fall 2020	-0.808***	-0.199***	0.077***
	(0.037)	(0.021)	(0.010)
Graduation cohort 2021	-0.053	-0.033	0.014
	(0.056)	(0.029)	(0.014)
At least one parent with university education	0.147***	-0.015	-0.000
	(0.056)	(0.029)	(0.014)
Missing information on parental education	0.109	-0.039	-0.018
	(0.099)	(0.049)	(0.024)
CATI interview	0.861***	0.224***	-0.137***
	(0.099)	(0.061)	(0.027)
Male	0.163***	0.214***	-0.185***
	(0.056)	(0.028)	(0.015)
GPA better than 2.5	0.197***	0.040	-0.010
	(0.052)	(0.027)	(0.014)
Missing information on GPA	-0.127	-0.092	-0.101*
	(0.272)	(0.116)	(0.060)
1st/2nd generation migrants	-0.222***	0.017	0.072***
	(0.069)	(0.034)	(0.017)
Missing information on migration status	-0.361***	-0.016	0.087***
	(0.124)	(0.061)	(0.030)
Dummy for being myopic	0.035	-0.027	0.006
	(0.081)	(0.039)	(0.020)
Risk aversion	-0.042***	-0.022***	0.014***
	(0.014)	(0.007)	(0.003)
Openness	-0.018	-0.049***	0.038***
	(0.022)	(0.011)	(0.005)
Conscientiousness	0.002	0.041**	0.005
	(0.035)	(0.017)	(0.009)
Extraversion	0.121***	-0.004	0.000
	(0.021)	(0.011)	(0.005)
Agreeableness	0.269***	0.079***	-0.032***
	(0.030)	(0.014)	(0.007)
Neuroticism	-0.190***	-0.120***	0.083***
	(0.024)	(0.012)	(0.006)
N observations	11091	11091	7394

Note: Robust standard errors in parentheses; Statistical significance: * p < 0.10, ** p < 0.05, *** p < 0.01. School fixed effects are included. As mental health problems were not included in the first survey wave, the baseline value in the anxiety and depression model is “Spring 2020 (Pre SC)” and not “Fall 2019”.
Data Source: Bero-Study wave 1 to 3

Table A4 Development of mental and physical health by graduation. Results from random effect growth curve models

	Life-satisfaction	Self-rated Health	Anxiety & depression risk
Spring 2020 (Pre SC)	-0.250***	-0.188***	
	(0.061)	(0.038)	
Spring 2020 (Post SC)	-0.363***	0.023	-0.041*
	(0.080)	(0.047)	(0.025)
Fall 2020	-0.657***	-0.097***	0.008
	(0.066)	(0.036)	(0.017)
Graduation cohort 2021	0.047	0.037	-0.033*
	(0.066)	(0.037)	(0.020)
Spring 2020 (Pre SC) x Graduation cohort 2021	-0.111	-0.063	
	(0.076)	(0.047)	
Spring 2020 (Post SC) x Graduation cohort 2021	-0.002	-0.045	-0.024
	(0.096)	(0.056)	(0.030)
Fall 2020 x Graduation cohort 2021	-0.228***	-0.155***	0.104***
	(0.079)	(0.044)	(0.021)
At least one parent with university education	0.146***	-0.015	-0.000
	(0.056)	(0.029)	(0.014)
Missing information on parental education	0.110	-0.039	-0.018
	(0.099)	(0.049)	(0.024)
CATI interview	0.865***	0.226***	-0.139***
	(0.099)	(0.061)	(0.027)
Male	0.163***	0.214***	-0.185***
	(0.056)	(0.028)	(0.015)
GPA better than 2.5	0.198***	0.040	-0.010
	(0.052)	(0.027)	(0.014)
Missing information on GPA	-0.129	-0.092	-0.100*
	(0.272)	(0.116)	(0.060)
1st/2nd generation migrants	-0.221***	0.017	0.072***
	(0.069)	(0.034)	(0.017)
Missing information on migration status	-0.362***	-0.016	0.087***
	(0.124)	(0.061)	(0.030)
Self-efficacy	0.930***	0.305***	-0.200***
	(0.080)	(0.039)	(0.019)
Grit	0.301***	-0.004	-0.027*
	(0.060)	(0.029)	(0.015)
Dummy for being myopic	0.035	-0.027	0.006
	(0.081)	(0.039)	(0.020)
Risk aversion	-0.042***	-0.022***	0.014***
	(0.014)	(0.007)	(0.003)
Openness	-0.017	-0.049***	0.038***
	(0.022)	(0.011)	(0.005)
Conscientiousness	0.002	0.041**	0.005
	(0.035)	(0.017)	(0.009)
Extraversion	0.121***	-0.004	0.000
	(0.021)	(0.011)	(0.005)
Agreeableness	0.269***	0.079***	-0.032***
	(0.030)	(0.014)	(0.007)
Neuroticism	-0.190***	-0.120***	0.083***
	(0.024)	(0.012)	(0.006)
Constant	2.630***	3.265***	0.700***
	(0.408)	(0.210)	(0.105)
N observations	11091	11091	7394

Note: Robust standard errors in parentheses; Statistical significance: * $p < 0.10$, ** $p < 0.05$, *** $p < 0.01$. School fixed effects are included. As mental health problems were not included in the first survey wave, the baseline value in the anxiety and depression model is “Spring 2020 (Pre SC)” and not “Fall 2019”.
Data Source: Bero-Study wave 1 to 3

Table A5 Placebo test: Immediate effects of school closures well-being: Results from difference-in-differences and OLS regressions

Panel A	Mean wave 1 Pre SC Placebo Group	Mean wave 1 Post SC Placebo Group	Mean wave 2 Pre SC Placebo Group	Mean wave 2 Post SC Placebo Group	DID in % of SE	p-value DID
Life satisfaction (0–10)	7.227	7.472	6.866	7.166	0.055	0.541
Self-rated health (1–5)	3.836	3.883	3.611	3.741	0.083	0.109

Panel B					Mean Diff. pre and post Placebo school closures	p-value
Mental Health Problems (0–1)			0.439	0.399	0.005	0.801
N persons	568	2,935	568	2,935		

Note: Panel A presents estimates in percent of standard deviation based on difference-in-difference regressions with federal state fixed effects. Panel B presents mean differences based on an OLS regression. Controls: school fixed effects, gender, migration status, parental education, school performance at wave 1, self-efficacy, Grit, Big Five personality traits, graduation cohort, risk aversion, time preferences, self-rated health, life satisfaction, and interview mode. SC = School Closures. In the placebo estimations, we define that the school closures happened one week earlier than the real school closures.
Data: BerO study wave 1 and 2.

Table A6 Effect of Interview Week on Life-satisfaction, Self-rated Health, and Mental Health Problems

	(1) Life satisfaction	(2) Self-rated Health	(3) Mental Health Problems (binary)	(4) HSCL-10 (continuous)
Interview week after start of the survey	-0.021	0.010	0.008	0.013**
	(0.016)	(0.010)	(0.004)	(0.005)
Dummy wave 3	-0.410***	-0.161***	0.128***	0.174***
	(0.058)	(0.034)	(0.014)	(0.017)
Individual fixed effects	Y	Y	Y	Y
Observations	2810	2810	2810	2810

Note. Outcomes: Life satisfaction (0 to 10); self-rated health (1 to 5); Mental Health Problems is a dummy for being above the clinical threshold for a high anxiety and depression risk (HSCL-10). HSCL-10 (continuous) is the original scale of the HSCL. The coefficients are estimated by an individual fixed effects model regressing the outcomes on Interview week after start of the survey. Statistical significance at * $p < 0.10$, ** $p < 0.05$, *** $p < 0.01$.

Table A7 Development of mental and physical health since fall 2019. Results from OLS, random effects and fixed effects models

Life satisfaction

	RE	OLS	FE
Survey waves (ref. wave 1)			
Survey wave 2	-0.3379***	-0.3386***	-0.3372***
	(0.0307)	(0.0307)	(0.0304)
Survey wave 3	-0.8081***	-0.8100***	-0.8062***
N Observations	11091	11091	11091

Self-rated Health

	RE	OLS	FE
Survey waves (ref. wave 1)			
Survey wave 2	-0.1453***	-0.1455***	-0.1449***
	(0.0185)	(0.0185)	(0.0183)
Survey wave 3	-0.2006***	-0.2013***	-0.1997***
N Observations	11091	11091	11091

HSCL: first difference estimator (aka a FE model)

	RE	OLS	FE
Survey waves (ref. wave 2)			
Survey wave 3	0.0995***	0.0996***	0.0989***
	(11.55)	(11.56)	(11.55)
N Observations	7394	7394	7394

Note. Standard Errors in parentheses; RE=Random effects Model; OLS= Ordinary Least Square Model, FE=Fixed Effects Modell; Statistical significance: * $p < 0.10$, ** $p < 0.05$, *** $p < 0.01$.

Figure A1 Immediate effects of school closures on mental and physical health: Results from difference-in-difference regressions

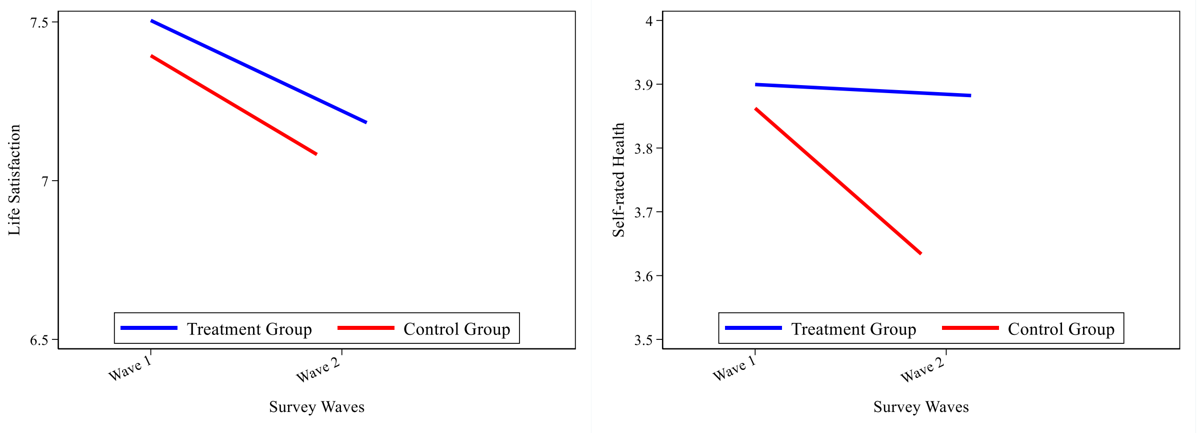

Comparison of analytical sample (truncated by transition) and untruncated sample

Figure A2 Immediate effects of school closures on mental and physical health: Results from difference-in-difference regressions. (Left hand side shows results from the manuscript. Right hand side shows results based on an untruncated sample, i.e., with individuals in gap years.)

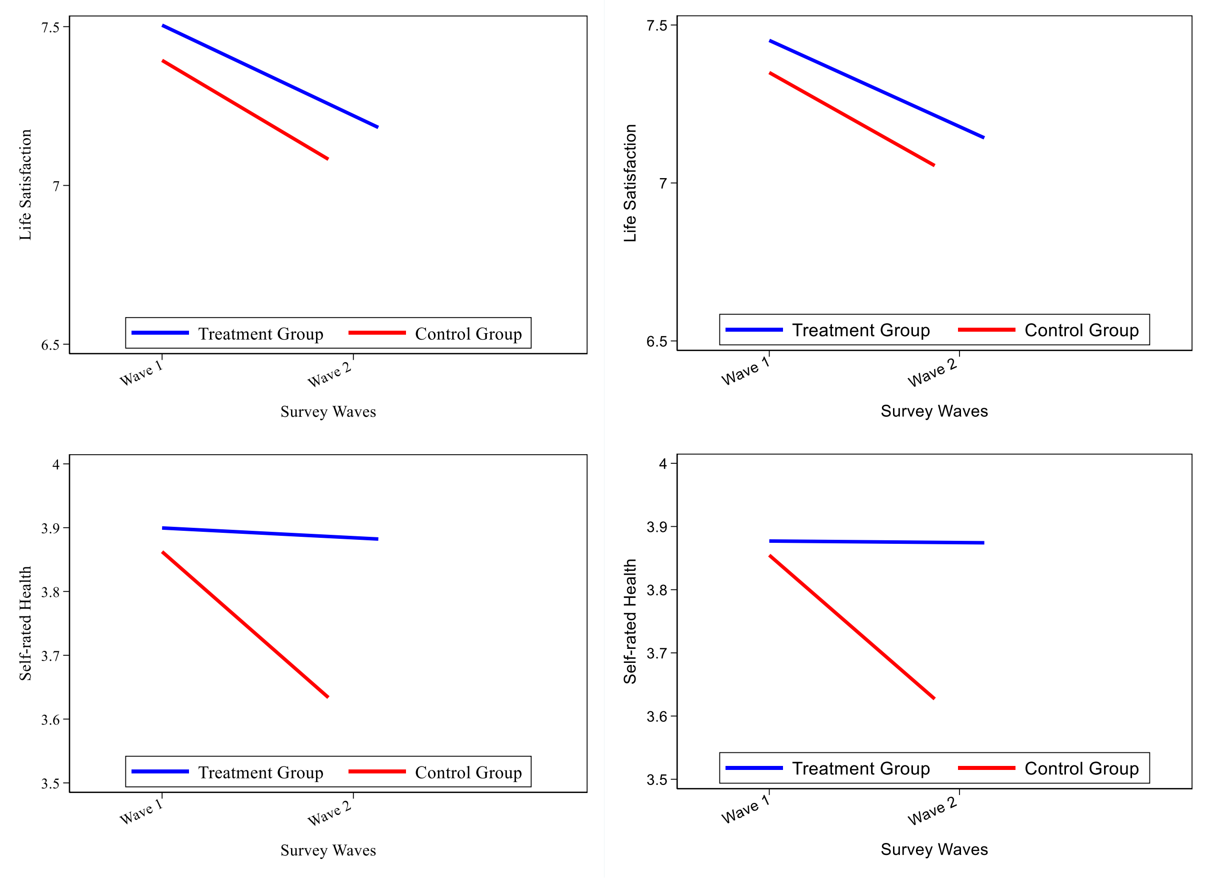

Figure A3 Development of mental and physical well-being since fall 2019. Results from random effect growth curve models. (Left hand side shows results from the manuscript. Right hand side shows results based on an untruncated sample, i.e., with individuals in gap years.)

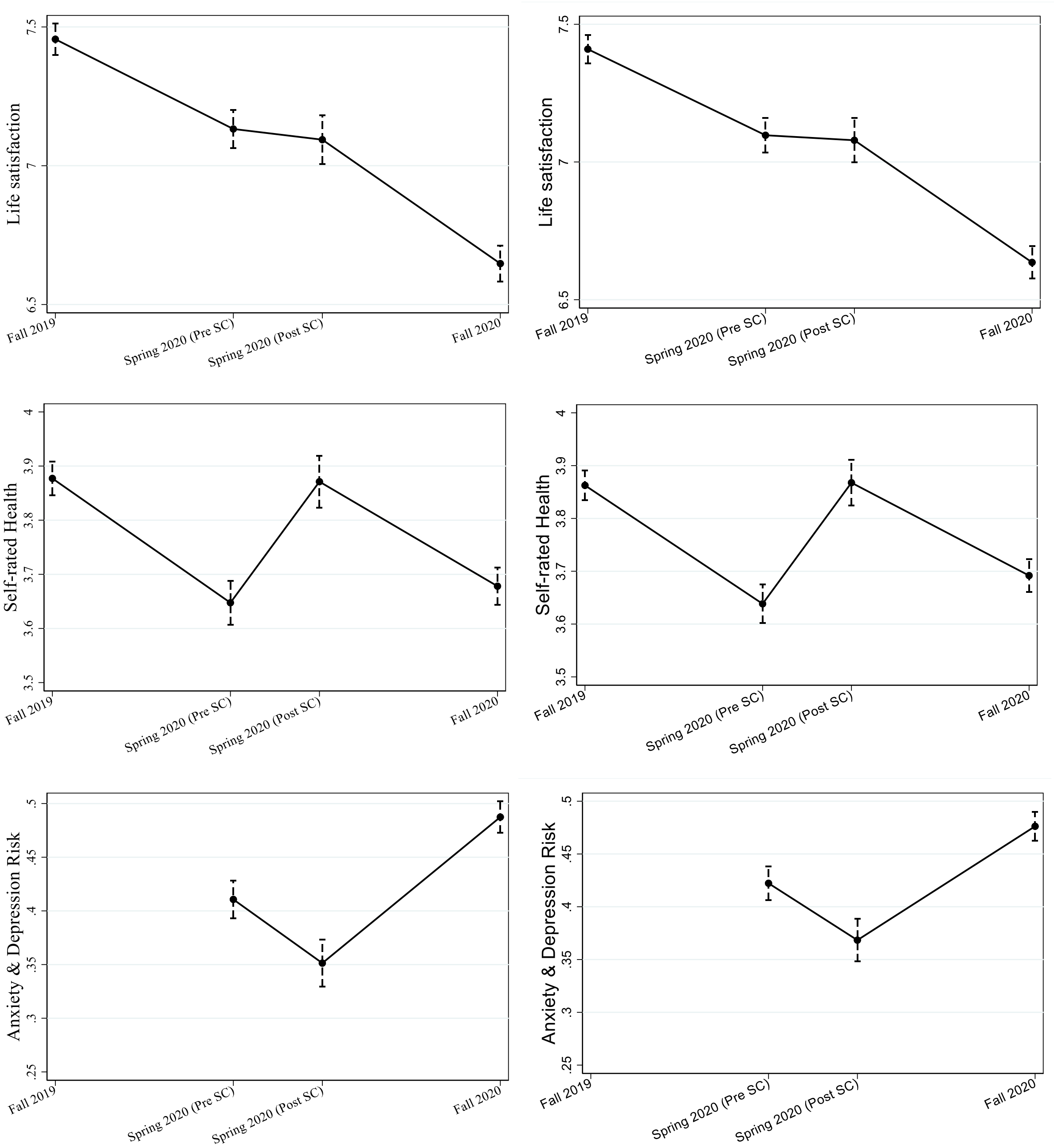

Figure A4 Development of mental and physical well-being by graduation cohort. Results from random effect growth curve models. (Left hand side shows results from the manuscript. Right hand side shows results based on an untruncated sample, i.e., with individuals in gap years.)

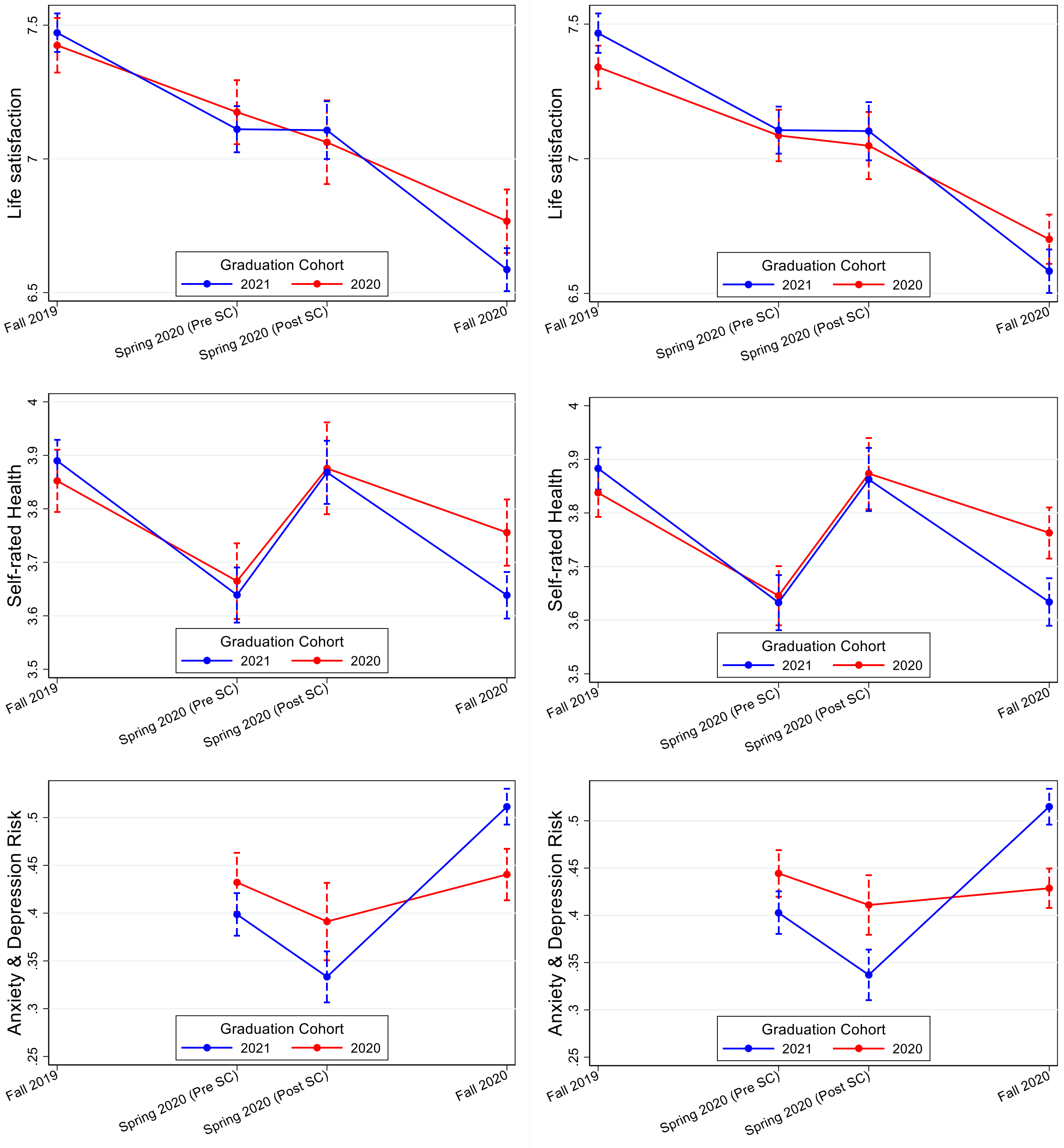

Figure A5 Difference Between Week of Survey Response in Wave 2 and 3 within Individuals

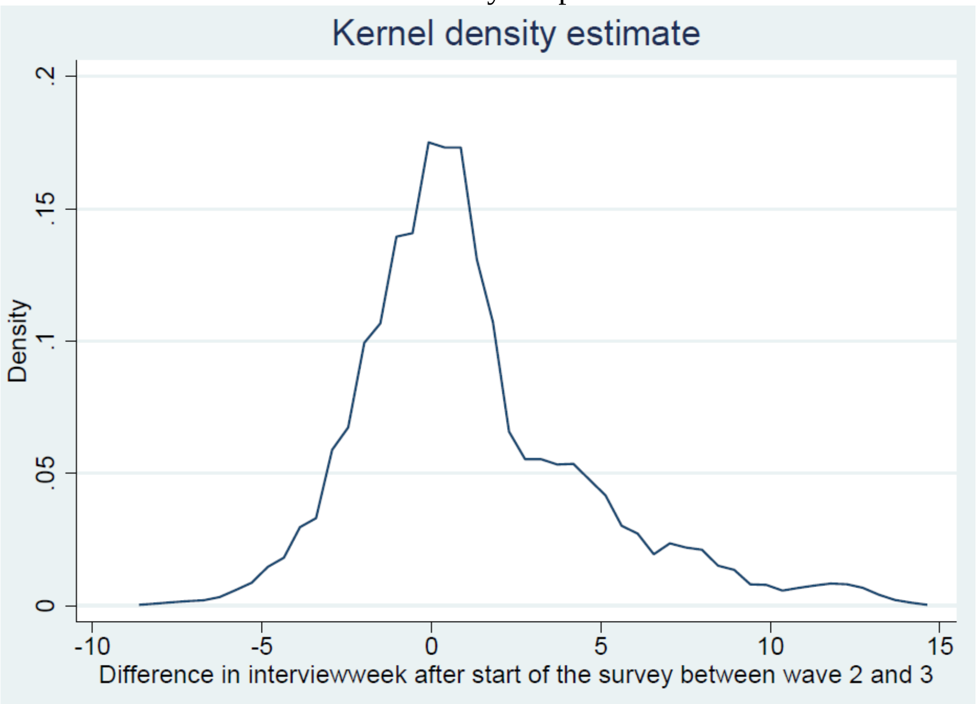

Note. Difference between week of survey respond in wave 2 and 3 within individuals. A negative value means that the individual responded earlier in wave 2 than wave 3. A zero value means that the individual answered the survey at the same week in wave 2 and 3. A positive value means that the individual responded later in wave 2 than wave 3.
